# Synthetic strategies for the preparation of γ-phostams: 1,2-azaphospholidine 2-oxides and 1,2-azaphospholine 2-oxides

**DOI:** 10.3762/bjoc.18.90

**Published:** 2022-07-22

**Authors:** Jiaxi Xu

**Affiliations:** 1 State Key Laboratory of Chemical Resource Engineering, Department of Organic Chemistry, College of Chemistry, Beijing University of Chemical Technology, Beijing 100029, People’s Republic of China; 2 College of Sciences, Henan Agricultural University, Zhengzhou 450002, People’s Republic of China

**Keywords:** azaphospholidine, azaphospholine, phosphonolactam, γ-phosphonolactam, phosphinolactam, γ-phosphinolactam, γ-phostam

## Abstract

γ-Phostams include γ-phosphonolactams and γ-phosphinolactams and their fused derivatives, phosphorus analogues of γ-lactams. They are 1,2-azaphospholidine 2-oxides and 1,2-azaphospholine 2-oxides and important biological five-membered azaphosphaheterocycles. They have been prepared through two major strategies of cyclizations and annulations. Cyclizations achieve ring construction through the formation of any bond in the ring, while annulations build the ring via [4 + 1] and [3 + 2] fashions with the simultaneous formation of two bonds. The review includes the synthesis of 1,2-azaphospholidine and 1,2-azaphospholine 2-oxides/sulfides and their fused derivatives.

## Introduction

**Figure 1:**
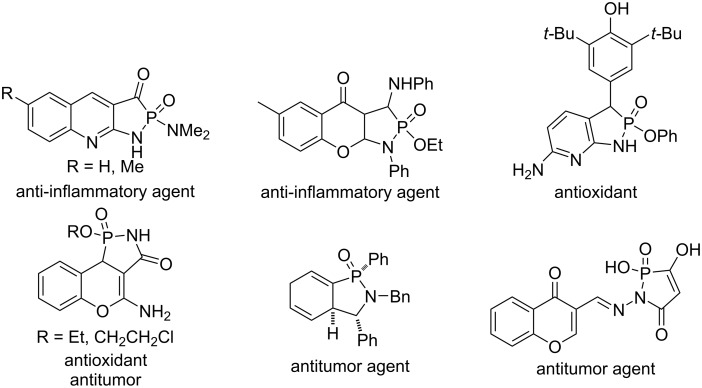
Biologically active 1,2-azaphospholine 2-oxide derivatives.

Phosphaheterocycles are a class of important organic compounds [1–8] and have been widely applied in agrochemicals, medicinal agents, and materials science [9–10]. They are also organic synthetic intermediates and building blocks [7,11,13]. 1,2-Azaphosphaheterocycle oxide derivatives, phosphonolactams and phosphinolactams, are phosphorus analogues of the corresponding lactams [11–14]. 1,2-Azaphospholidine 2-oxides and 1,2-azaphospholine 2-oxides, also called γ-phosphonolactams and γ-phosphinolactams, and their fused derivatives are important five-membered 1,2-azaphosphaheterocyclic derivatives. They are γ-phostams, phosphorus analogues of γ-lactams, showing various biological activities, such as anti-inflammatory [15–16], antioxidant [17–18], and antitumor [18–20] (Figure 1).

**Figure 2:**
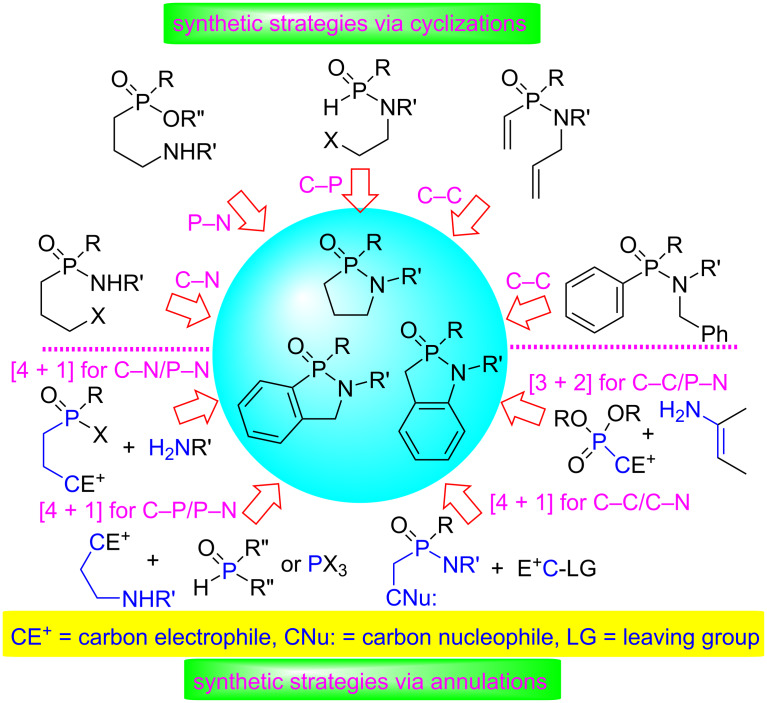
Diverse synthetic strategies for the preparation of 1,2-azaphospholidine and 1,2-azaphospholine 2-oxide derivatives.

Various synthetic methods of 1,2-azaphospholidine 2-oxide and 1,2-azaphospholine 2-oxide derivatives have been developed to date. Two major synthetic strategies are cyclization reactions and annulation reactions. The cyclization reactions have been applied in the construction of any ring bonds of 1,2-azaphospholidine and 1,2-azaphospholine rings, while [4 + 1] and [3 + 2] annulations are alternative routes for the formation of 1,2-azaphospholidine and 1,2-azaphospholine rings (Figure 2). This review includes the synthesis of 1,2-azaphospholidine and 1,2-azaphospholine 2-oxides/sulfides and their fused derivatives.

## Review

### Synthesis of 1,2-azaphospholidine 2-oxide derivatives via cyclization

Various cyclization strategies have been developed for the synthesis of 1,2-azaphospholidine 2-oxides/sulfides and their fused derivatives. The 1,2-azaphospholidine 2-oxide/sulfide derivatives have been prepared by construction of any of their ring bonds.

#### Synthesis via C–N bond formation

**Scheme 1:**
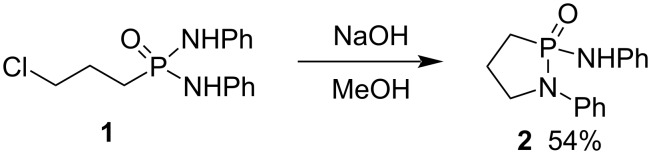
Synthesis of 1-phenyl-2-phenylamino-γ-phosphonolactam (**2**) from *N*,*N*’-diphenyl 3-chloropropylphosphondiamide (**1**).

In 1962, Helferich and Curtius reported the first synthesis of a 1,2-azaphospholidine 2-oxide (γ-phosphonolactam) from *N*,*N*’-diphenyl 3-chloropropylphosphondiamide (**1**), which was cyclized to 1-phenyl-2-phenylamino-γ-phosphonolactam (**2**) in the presence of NaOH in methanol via the C–N bond formation (Scheme 1) [21].

**Scheme 2:**
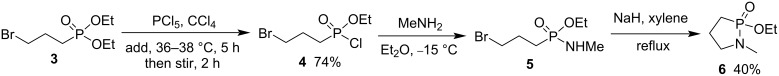
Synthesis of 2-ethoxy-1-methyl-γ-phosphonolactam (**6**) from ethyl *N*-methyl-(3-bromopropyl)phosphonamidate (**5**).

In 1974, the strategy was applied for the synthesis of 2-ethoxy-1-methyl-γ-phosphonolactam (**6**) for potential insecticides. Diethyl 3-bromopropylphosphonate (**3**) was first monochlorinated with phosphorus pentachloride in carbon tetrachloride followed by aminolysis with methylamine, affording ethyl *N*-methyl-(3-bromopropyl)phosphonamidate (**5**). Compound **5** was then treated with NaH in refluxing xylene to give the desired 2-ethoxy-1-methyl-γ-phosphonolactam (**6**) in approximate 40% yield (Scheme 2) [22].

**Scheme 3:**
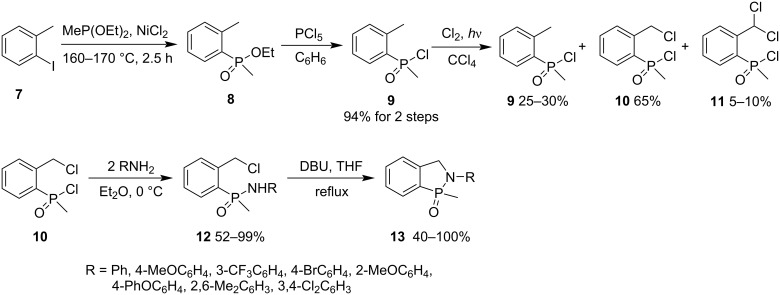
Synthesis of 2-aryl-1-methyl-2,3-dihydrobenzo[*c*][1,2]azaphosphole 1-oxides **13** from *N*-aryl-2-chloromethylphenyl(methyl)phosphinamides **12**.

In 1981, Miles and co-workers synthesized 2-aryl-1-methyl-2,3-dihydrobenzo[*c*][1,2]azaphosphole 1-oxides **13** from 2-iodotoluene (**7**). The reaction of 2-iodotoluene (**7**) and diethyl methylphosphonite gave ethyl 2-methylphenyl(methyl)phosphinate (**8**) in an excellent yield under the catalysis of anhydrous NiCl_2_ (Scheme 3) [23]. Ethyl 2-methylphenyl(methyl)phosphinate (**8**) was converted into 2-methylphenyl(methyl)phosphinic chloride (**9**) in 94% yield by treatment with excess PCl_5_. However, the radical chlorination of 2-methylphenyl(methyl)phosphinic chloride (**9**) gave the desired 2-chloromethylphenyl(methyl)phosphinic chloride (**10**) in 65% yield with unreacted starting **9** in 25–30%, and the dichlorinated product **11** in 5–10%. The reaction of 2-chloromethylphenyl(methyl)phosphinic chloride (**10**) with amines generated *N*-aryl-2-chloromethylphenyl(methyl)phosphinamides **12** in 52–99% yields, which were further treated with DBU in refluxing THF, affording 2-aryl-1-methyl-2,3-dihydrobenzo[*c*][1,2]azaphosphole 1-oxides **13** in 40–100% yield (Scheme 3) [23]. This is a general method to synthesize 1,2-azaphospholidine 2-oxide derivatives **13**.

**Scheme 4:**
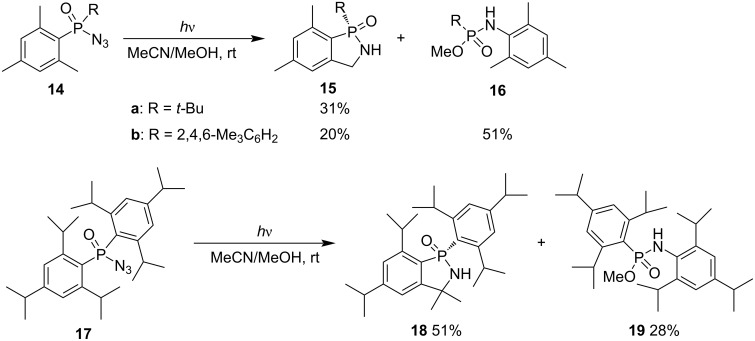
Synthesis of 2,3-dihydrobenzo[*c*][1,2]azaphosphole 1-oxides from alkylarylphosphinyl or diarylphosphinyl azides via an intramolecular nitrene C–H insertion.

Arylphosphinyl azides generate arylphosphinyl nitrenes under photoirradiation. The phosphinyl nitrenes underwent an intramolecular insertion into the *ortho* C–H bond of the aryl group accompanied with the Curtius-like rearrangement as well [24]. Both monomesityl and dimesitylphosphinyl azides **14** generated 2,3-dihydrobenzo[*c*][1,2]azaphosphole 1-oxides **15** in 31% and 20%, respectively, via an intramolecular nitrene C–H insertion, for dimesitylphosphinyl azide (**14b**), with 51% yield of phosphonamidate **16** as byproduct. Bis(2,4,6-triisopropylphenyl)phosphinyl azide (**17**) gave the corresponding 2,3-dihydrobenzo[*c*][1,2]azaphosphole 1-oxide **18** in 51% and phosphonamidate **19** in 28% yield (Scheme 4). The synthetic method was applied occasionally [24].

**Scheme 5:**
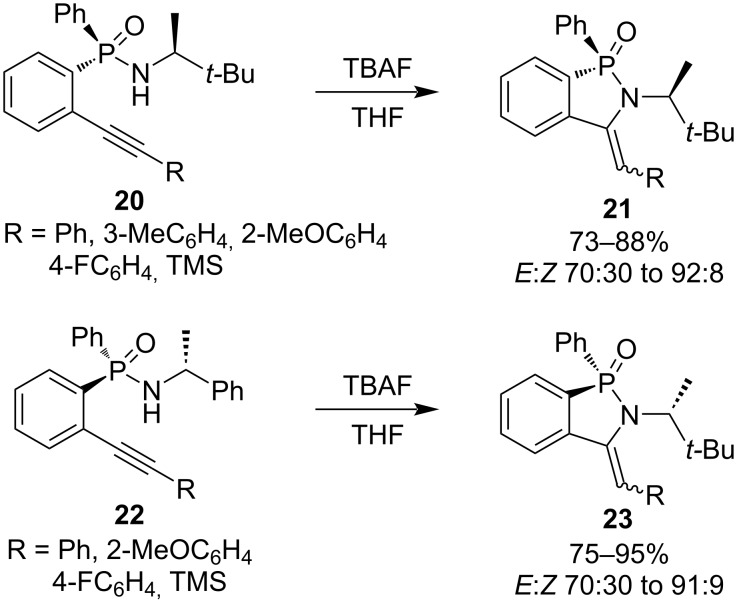
Synthesis of 3-arylmethylidene-2,3-dihydrobenzo[*c*][1,2]azaphosphole 1-oxides via the TBAF-mediated cyclization of *N*-alkyl-*P*-(2-ethynylaryl)-*P*-phenylphosphinamides.

*P*-Stereogenic 3-arylmethylidene-2,3-dihydrobenzo[*c*][1,2]azaphosphole 1-oxides **21** and **23** were prepared in good to excellent yields with moderate to good *E*/*Z* selectivities via the TBAF-mediated cyclization of enantiopure *N*-alkyl-*P*-(2-ethynylaryl)-*P*-phenylphosphinamides **20** and **22**. The synthetic strategy provided optically active 2,3-dihydrobenzo[*c*][1,2]azaphosphole 1-oxide derivatives **21** and **23** (Scheme 5) [25].

**Scheme 6:**
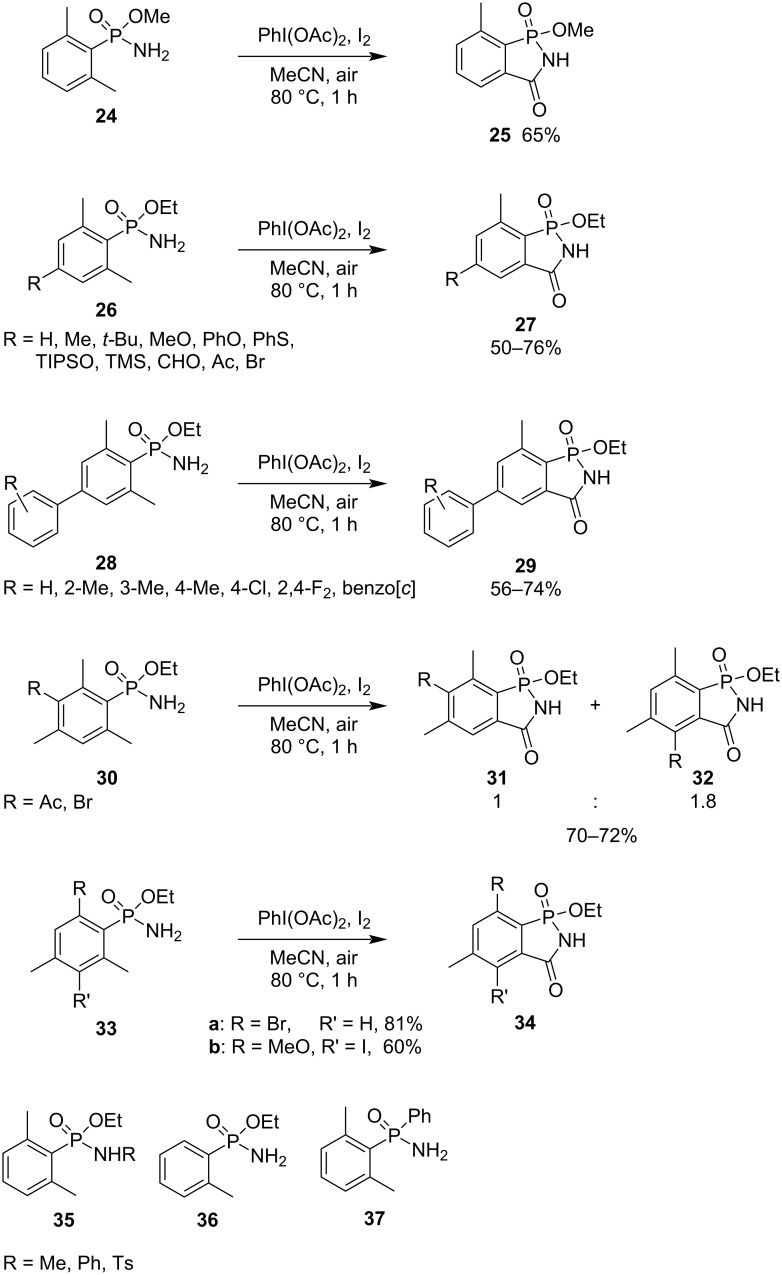
Synthesis of 2-hydrobenzo[*c*][1,2]azaphosphol-3-one 1-oxides via the metal-free intramolecular oxidative C–H bond formation of alkyl 6-substituted 2-methylphenylphosphonamidates.

The metal-free intramolecular oxidative C–H bond amidation of methyl and ethyl 2,6-dimethylphenylphosphonamidates **24**, **26**, and **28** is an interesting strategy for the synthesis of 1-methoxy/ethoxy-7-methyl-2-hydrobenzo[*c*][1,2]azaphosphol-3-one 1-oxide derivatives **25**, **27**, and **29** in satisfactory to good yields (Scheme 6) [26]. The 2,6-dimethylphenyl (**26**) and 4-aryl-2,4-dimethylphenyl (**28**) groups can be substituted by various functional groups. The same reaction using ethyl 3-substituted 2,4,6-trimethylphenylphosphonamidates **30** generated two regioisomeric 5,7-dimethyl-2-hydrobenzo[*c*][1,2]azaphosphol-3-one 1-oxides **31** and **32**. Also, the ethyl 6-bromo-2,4-dimethylphenylphosphonamidate (**33a**) and the electron-rich 6-methoxy substrate **33b** gave the corresponding 7-bromo and 4-iodo products **34a** and **34b**. However, ethyl *N*-substituted 2,6-dimethylphenylphosphonamidates **35**, ethyl 2-methylphenylphosphonamidate (**36**), and ethyl *P*-phenyl 2,6-dimethylphenylphosphinamide (**37**) did not undergo the reaction, showing a limited application of the synthetic strategy (Scheme 6) [26]. The products are carboxylic phosphonic imides.

**Scheme 7:**
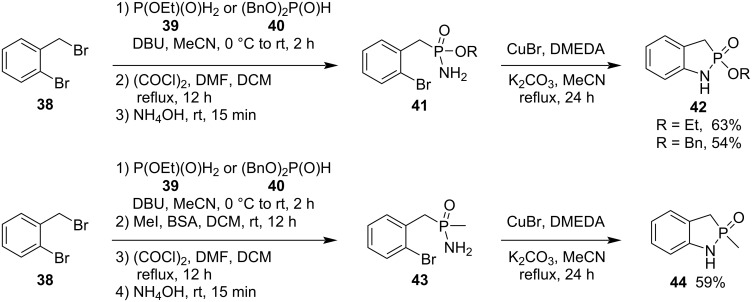
Synthesis of 1,3-dihydrobenzo[*d*][1,2]azaphosphole 2-oxides **42** and **44** from ethyl/benzyl 2-bromobenzylphosphonamidates **41** and *P*-(2-bromobenzyl)-*P*-(methyl)phosphinamide (**43**).

In 2021, Montchamp and co-workers synthesized 1,3-dihydrobenzo[*d*][1,2]azaphosphole 2-oxides **42** and **44** in moderate 54–63% yields via the intramolecular copper-catalyzed cross-coupling of ethyl/benzyl 2-bromobenzylphosphonamidates **41** or *P*-(2-bromobenzyl)-*P*-(methyl)phosphinamide (**43**) as a key step. They were prepared from 2-bromobenzyl bromide (**38**) via three and four steps, respectively (Scheme 7) [27].

#### Synthesis via P–N bond formation

**Scheme 8:**
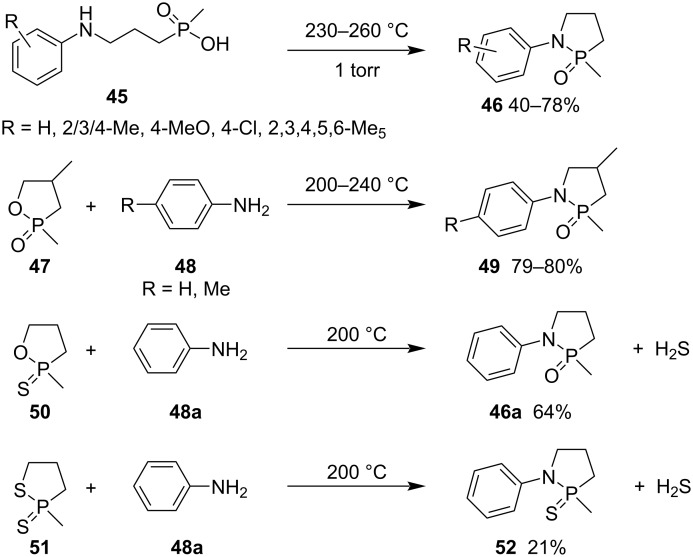
Synthesis of azaphospholidine 2-oxides/sulfide from 1,2-oxaphospholane 2-oxides/sulfides and 1,2-thiaphospholane 2-sulfide with anilines.

Since 1980, new strategies for the synthesis of γ-phosphonolactams and γ-phosphinolactams have been explored via cyclization by P–N bond formation [28]. Kleiner prepared 1-aryl-2-methyl-1,2-azaphospholidine 2-oxides **46** in 40–78% yields by heating 3-arylaminopropyl(methyl)phosphinic acids **45** under reduced pressure. The reactions of 1,2-oxaphospholane 2-oxides **47**/2-sulfides **50** and anilines **48** generated 1-aryl-2-methyl-1,2-azaphospholidine 2-oxides **49** and **46a** as well, while the reaction of 1,2-thiaphospholane 2-sulfide (**51**) with aniline (**48a**) gave azaphospholidine 2-sulfide **52** in only 21% yield by heating at 200 °C for 60 h (Scheme 8) [28].

**Scheme 9:**
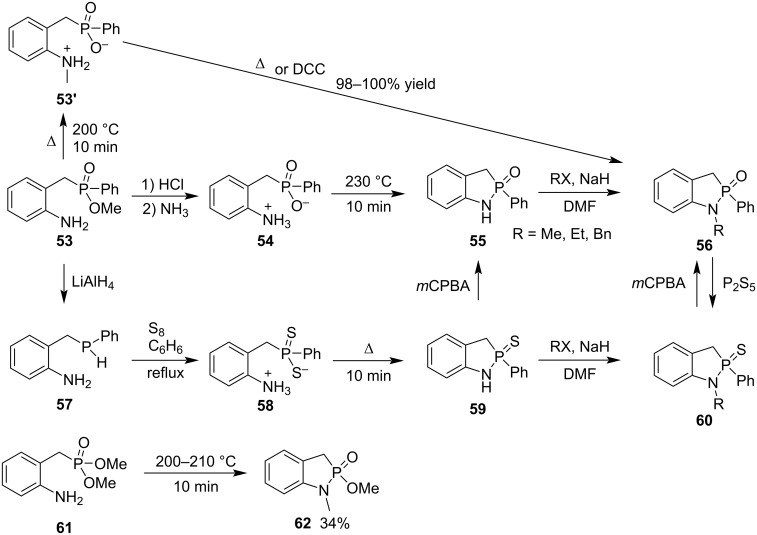
Synthesis of 1,3-dihydrobenzo[*d*][1,2]azaphosphole 2-oxides/sulfides from 2-aminobenzyl(phenyl)phosphinic acid.

In 1982, Collins and co-workers prepared both 1,3-dihydrobenzo[*d*][1,2]azaphosphole 2-oxides **56** and 2-sulfide **60** through heating zwitterionic 2-aminobenzyl(phenyl)phosphinic acid **54** and 2-aminobenzyl(phenyl)dithiophosphinic acid **58**, respectively. On the other hand, methyl 2-aminobenzyl(phenyl)phosphinate (**53**) was hydrolyzed under acidic conditions and neutralized with ammonia to give zwitterionic 2-aminobenzyl(phenyl)phosphinic acid **54**. It was heated at 230 °C under reduced pressure to generate 2-phenyl-1,3-dihydrobenzo[*d*][1,2]azaphosphole 2-oxide (**55**), which was further alkylated with alkyl halides in the presence of NaH, affording 1-alkyl-2-phenyl-1,3-dihydrobenzo[*d*][1,2]azaphosphole 2-oxides **56**. Alternatively, under heating, methyl 2-aminobenzyl(phenyl)phosphinate (**53**) underwent a transmethylation from the O to N atom to generate the zwitterionic 2-((methylamino)benzyl)(phenyl)phosphinic acid **53’**, which was further converted into 1-methyl-2-phenyl-1,3-dihydrobenzo[*d*][1,2]azaphosphole 2-oxide (**56a**, R = Me) in excellent yield under heating or treatment with DCC. On the other way, methyl (2-aminobenzyl)(phenyl)phosphinate (**53**) was reduced with lithium aluminum hydride to 2-aminobenzyl(phenyl)phosphine (**57**). It was oxidized with sulfur to give zwitterionic 2-aminobenzyl(phenyl)dithiophosphinic acid (**58**), which underwent thermal elimination of hydrogen sulfide to yield 2-phenyl-1,3-dihydrobenzo[*d*][1,2]azaphosphole 2-sulfide (**59**). Sulfide **59** was similarly transformed to 1-alkyl-2-phenyl-1,3-dihydrobenzo[*d*][1,2]azaphosphole 2-sulfides **60** by treatment with alkyl halides in the presence of NaH. All 1,3-dihydrobenzo[*d*][1,2]azaphosphole 2-sulfides **60** were converted into the corresponding 1,3-dihydrobenzo[*d*][1,2]azaphosphole 2-oxides **56** by oxidation with *m*CPBA, while treatment of 1,3-dihydrobenzo[*d*][1,2]azaphosphole 2-oxides **56** with phosphorus pentasulfide transformed them back to 1,3-dihydrobenzo[*d*][1,2]azaphosphole 2-sulfides **60**. Heating dimethyl 2-aminobenzylphosphonate (**61**) generated 1-methyl-2-methoxy-1,3-dihydrobenzo[*d*][1,2]azaphosphole 2-oxide (**62**) in 34% yield via transmethylation and intramolecular cyclocondensation (Scheme 9) [29].

**Scheme 10:**
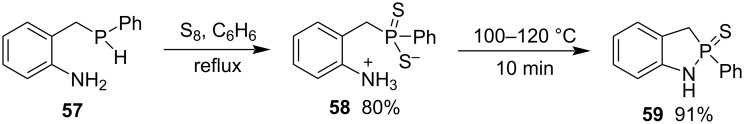
Synthesis of 1,3-dihydrobenzo[*d*][1,2]azaphosphole 2-sulfide (**59**) from zwitterionic 2-aminobenzyl(phenyl)dithiophosphinic acid (**58**).

In 1983, Collins and co-workers prepared 2-phenyl-1,3-dihydrobenzo[*d*][1,2]azaphosphole 2-sulfide (**59**) following the similar procedure. They first oxidized 2-aminobenzyl(phenyl)phosphine (**57**) with two atom-equivalents of sulfur in refluxing benzene, affording the zwitterionic 2-aminobenzyl(phenyl)dithiophosphinic acid **58** in 80% yield. It was converted to 2-phenyl-1,3-dihydrobenzo[*d*][1,2]azaphosphole 2-sulfide (**59**) in 91% yield by heating at 100–120 °C under vacuum (Scheme 10) [30].

**Scheme 11:**
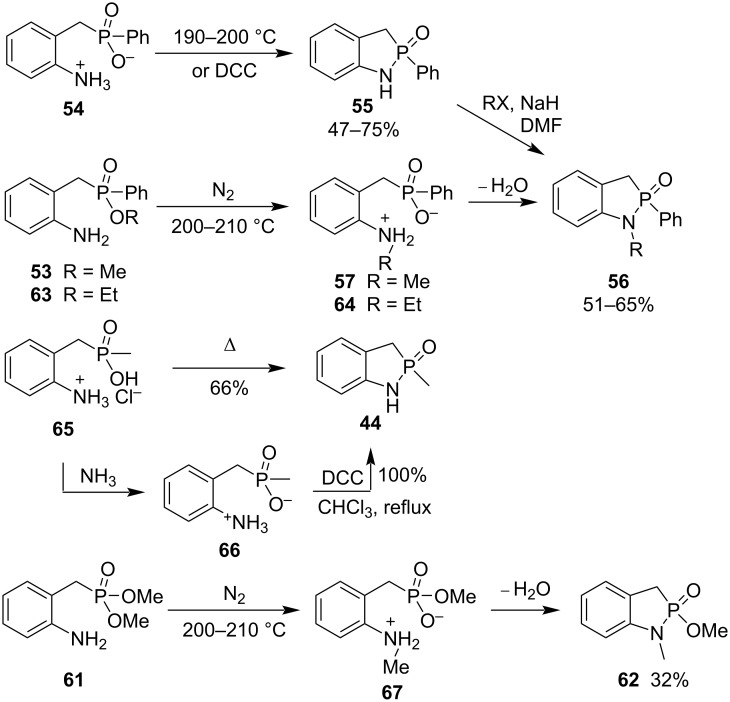
Synthesis of 1,3-dihydrobenzo[*d*][1,2]azaphosphole 2-oxides from 2-aminobenzyl(methyl/phenyl)phosphinic acids, alkyl 2-aminobenzyl(phenyl)phosphinates, and dimethyl 2-aminobenzylphosphonate (**61**).

In the same year, they also realized the synthesis of 2-phenyl-1,3-dihydrobenzo[*d*][1,2]azaphosphole 2-oxide (**55**) in 75% yield by heating at 190–200 °C and in 47% yield by DCC condensation of compound **54** – the oxygen analogue of 2-aminobenzyl(phenyl)dithiophosphinic acid **58**. The cyclodehydration method was more efficient than the DCC coupling one. The similar treatment of alkyl 2-aminobenzyl(phenyl)phosphinates **53** and **63** gave 1-alkyl-2-phenyl-1,3-dihydrobenzo[*d*][1,2]azaphosphole 2-oxides **56** in 51–65% yield through the alkyl transfer from the O to the N atom followed by dehydration under heating. The direct heating of 2-aminobenzyl(methyl)phosphinic acid hydrochloric acid salt (**65**) generated 2-methyl-1,3-dihydrobenzo[*d*][1,2]azaphosphole 2-oxide (**44**) in 66% yield. However, when compound **65** was carefully neutralized with ammonia to give the zwitterionic 2-aminobenzylmethylphosphinic acid (**66**), the conversion into 2-methyl-1,3-dihydrobenzo[*d*][1,2]azaphosphole 2-oxide (**44**) proceeded in a quantitative yield in the presence of DCC in refluxing chloroform. Also, dimethyl 2-aminobenzylphosphonate (**61**) was transformed to 1-methyl-2-methoxy-1,3-dihydrobenzo[*d*][1,2]azaphosphole 2-oxide (**62**) via a similar alkyl transfer from the O to N atom followed by dehydration under heating (Scheme 11) [31].

**Scheme 12:**
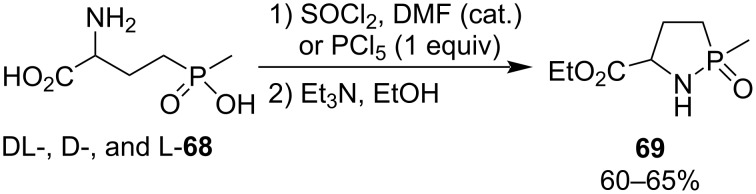
Synthesis of ethyl 2-methyl-1,2-azaphospholidine-5-carboxylate 2-oxide **69** from 2-amino-4-(hydroxy(methyl)phosphoryl)butanoic acids **68**.

Natchev prepared racemic and optically active ethyl 2-methyl-1,2-azaphospholidine-5-carboxylate 2-oxide (**69**) as cyclic analogue of the herbicide phosphinothricin (glufosinate, ᴅʟ-**68**) from ᴅʟ-, ᴅ-, and ʟ-2-amino-4-(hydroxy(methyl)phosphoryl)butanoic acid (**68**) by treatment with phosphorus pentachloride or thionyl chloride, respectively, in the presence of triethylamine when he synthesized phosphonopeptides. Similar yields were obtained for these two different chlorinating reagents. Ethyl 2-methyl-1,2-azaphospholidine-5-carboxylate 2-oxide (**69**) was sensible to glutaminase, an enzyme that could ring-open 1,2-azaphospholidine-5-carboxylate 2-oxides (Scheme 12) [32].

**Scheme 13:**
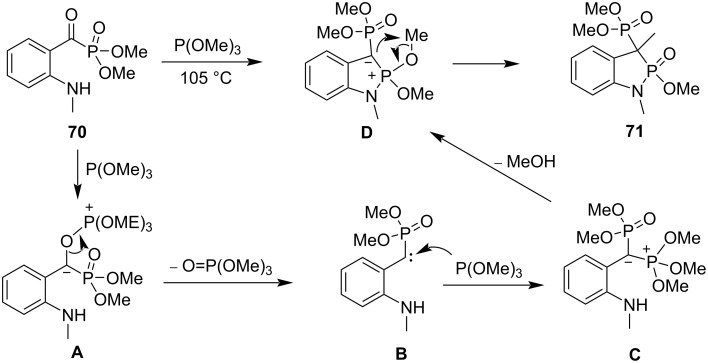
Synthesis of 2-methoxy-1,3-dihydrobenzo[*d*][1,2]azaphosphole 2-oxide **71** from dimethyl 2-(methylamino)benzoylphosphonate (**70**) and trimethyl phosphite.

Griffiths and co-workers mentioned the synthesis of dimethyl (2-methoxy-1,3-dimethyl-2-oxido-1,3-dihydrobenzo[*d*][1,2]azaphosphol-3-yl)phosphonate (**71**) from the reaction of dimethyl 2-(methylamino)benzoylphosphonate (**70**) and trimethyl phosphite at 105 °C through an ylide intermediate **D**. The ylide **D** was generated via deoxygenation of benzoylphosphonate **70** with trimethyl phosphite to form a carbene intermediate **B**, and trimethyl phosphite nucleophilic attacking to the carbene **B** followed by an intramolecular displacement. The yield was not reported (Scheme 13) [33].

#### Synthesis via P–C bond formation

**Scheme 14:**
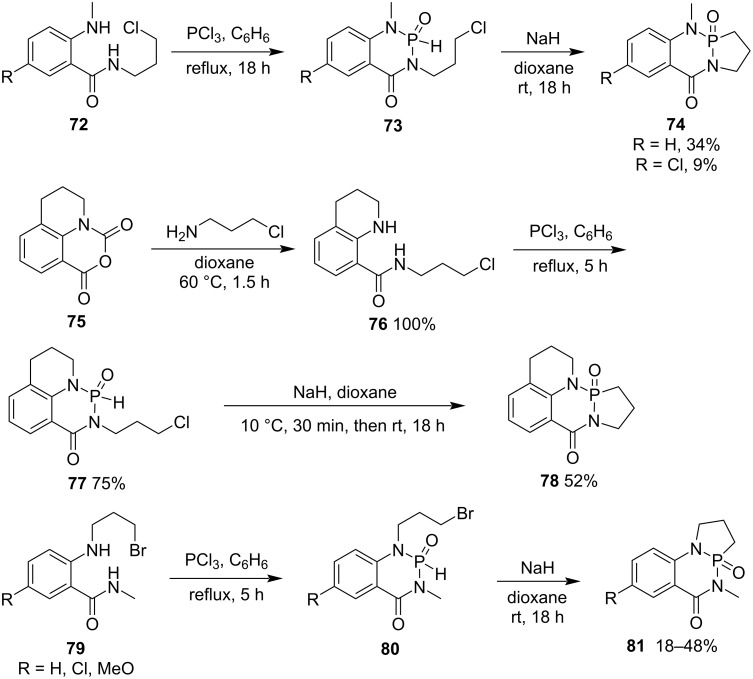
Synthesis of tricyclic γ-phosphonolactams via formation of the P–C bond.

In 1979, Coppola at Sandoz, Inc. reported an alternative strategy for the synthesis of tricyclic γ-phosphonolactams **74**, **78**, and **81** from *N*-(3-chloropropyl)-2-methylaminobenzamides **72**, **76** and *N*-methyl-2-(3-bromopropylamino)benzamides **79** via the P–C bond formation as the crucial step. *N*-(3-Chloropropyl)-2-methylaminobenzamides **72** and **76** and *N*-methyl-2-(3-bromopropylamino)benzamides **79** were first treated with phosphorus trichloride followed by intramolecular cyclization in the presence of sodium hydride in dioxane, affording tricyclic γ-phosphonolactams **74**, **78**, and **81** in low to moderate yields (Scheme 14) [34].

**Scheme 15:**
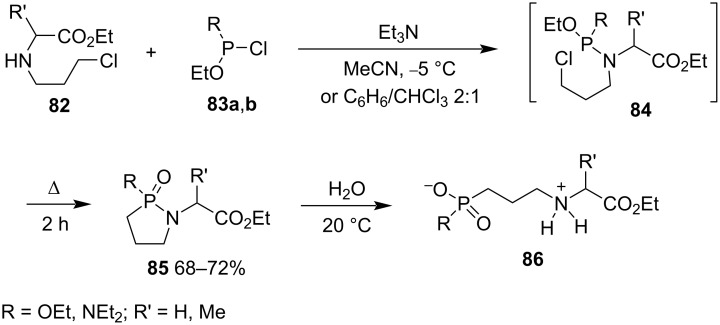
Synthesis of γ-phosphonolactams **85** from ethyl 2-(3-chloropropyl)aminoalkanoates with diethyl chlorophosphite and ethyl *N,N*-diethylchlorophosphamidite.

In 2005, Aladzheva and co-workers prepared γ-phosphonolactams **85** from the substitution of ethyl 2-(3-chloropropyl)aminoalkanoates **82** derived from glycine and ᴅʟ-alanine ethyl esters and diethyl chlorophosphite (**83a**, R = EtO) or ethyl *N,N*-diethylchlorophosphamidite (**83b**, R = NEt_2_) followed by an intramolecular cyclization via an intramolecular Arbuzov reaction under heating [35]. The obtained γ-phosphonolactams **85** were further hydrolyzed into 2-(3-phosphonopropyl)aminoalkanoic acids **86** (Scheme 15) [35–36]. This is a convenient way to synthesize γ-phosphonolactams **85**.

**Scheme 16:**
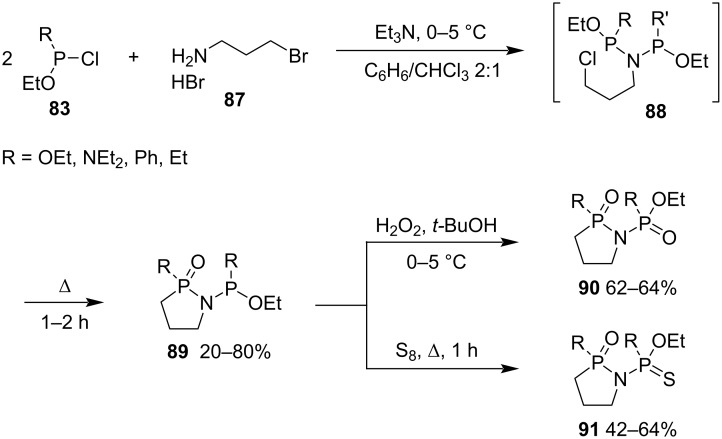
Synthesis of *N*-phosphoryl- and *N*-thiophosphoryl-1,2-azaphospholidine 2-oxides **90**/2-sulfides **91** from 3-bromopropylamine hydrogen bromide and chloroethoxyphosphine derivatives.

They further extended their method to synthesize cyclic *O*,*O*- and *O*,*S*-bidentate ligands with a P–N–P backbone. The substitution reaction of 3-bromopropylamine hydrogen bromide (**87**) and chloroethoxyphosphine derivatives **83** followed by an intramolecular cyclization via the intramolecular Arbuzov reaction under heating generated 1,2-azaphospholidine 2-oxides **89**. Compounds **89** were further transformed into *N*-phosphoryl- and *N*-thiophosphoryl-1,2-azaphospholidine 2-oxides **90**/2-sulfides **91** via oxidation with hydrogen peroxide and sulfurization with sulfur, respectively (Scheme 16) [37–38].

#### Synthesis via formation of the C–C bond neighboring at the ring phosphorus atom

**Scheme 17:**
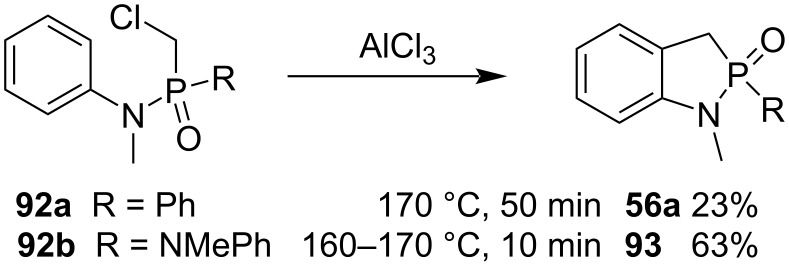
Synthesis of 1-methyl-1,3-dihydrobenzo[*d*][1,2]azaphosphole 2-oxides **56a** and **93** from *P*-(chloromethyl)amide precursors **92a** and **92b** through intramolecular Friedel–Crafts alkylation.

In 1984, Collins and co-workers attempted the synthesis of benzo-γ-phosphonolactams **56a** and **93** from (chloromethyl)(phenyl)-*N*-methyl-*N*-phenylphosphinamide (**92a**) and chloromethyl *N,N’*-dimethyl-*N,N*’-diphenylphosphondiamide (**92b**) via an intramolecular Friedel–Crafts alkylation. Although they tried several different amide derivatives, only phosphinamide **92a** and phosphonic diamide **92b** gave the corresponding 1-methyl-1,3-dihydrobenzo[*d*][1,2]azaphosphole 2-oxides **56a** in 23% and **93** in 63% yields, respectively, showing the limited scope of the synthetic method (Scheme 17) [39].

Ring-closing metathesis (RCM) is an efficient strategy for the construction of cyclic compounds via the formation of a C=C bond [40–41], which can be reduced to the C–C bond.

**Scheme 18:**
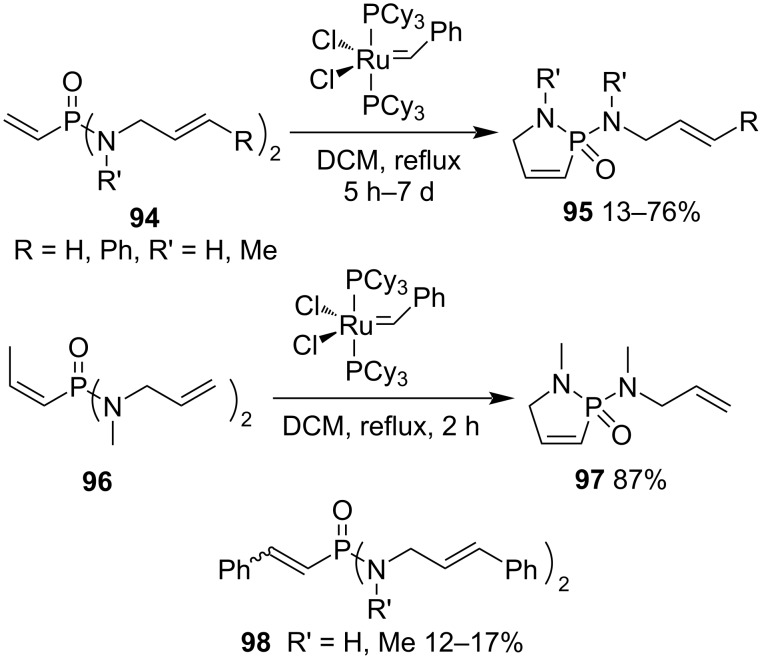
Synthesis of 2-allylamino-1,5-dihydro-1,2-azaphosphole 2-oxides from *N,N’*-diallyl-vinylphosphonodiamides.

2-Allylamino-1,5-dihydro-1,2-azaphosphole 2-oxide derivatives **95** were prepared in 13–76% yield from *N,N’*-diallyl-vinylphosphonodiamides **94** via the Grubbs ruthenium-catalyzed RCM. *N*,*N’*-Dicinnamyl-*N,N*’-dimethyl-vinylphosphonodiamide (**94a**) (R = R’ = H) generated the 2-(*N*-methyl-*N*-cinnamylamino)-1,5-dihydro-1,2-azaphosphole 2-oxide (**95a**) in the lowest yield. The (*E*)-prop-1-enylphosphonodiamide **96** also underwent the RCM well. However, *N*,*N’*-dicinnamyl-vinylphosphonodiamides **94b**,**c** (R’ = H, Me, R = Ph) always generated the corresponding *N*,*N’*-dicinnamyl-styrylphosphonodiamides **98** in 12–17% yields as byproducts via the RCM with the generated styrene in the reaction mixture (Scheme 18) [42].

**Scheme 19:**
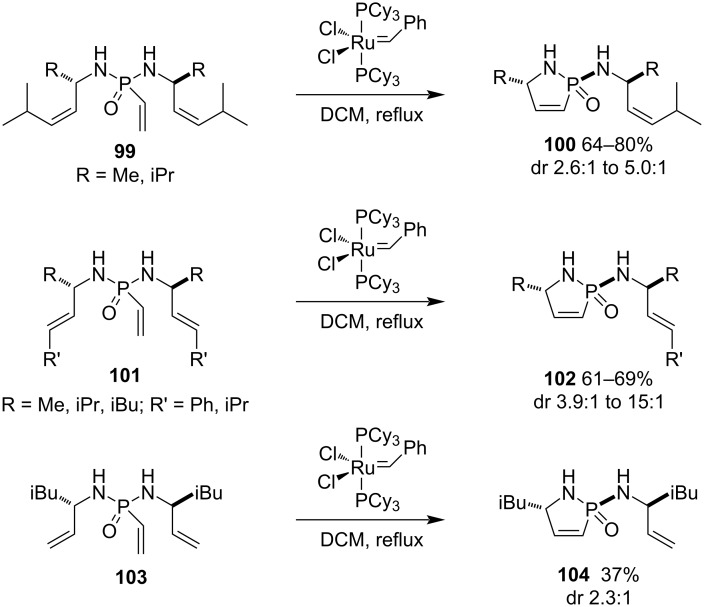
Diastereoselective synthesis of 2-allylamino-1,5-dihydro-1,2-azaphosphole 2-oxides from *N,N’*-diallyl-vinylphosphonodiamides.

One year later, the same group investigated the influence of the double bond geometry and the substitution pattern on the alkene. The results indicated that the double bond geometry plays a moderate role, while the bulkiness of the R group and the nature of its terminal substituents (aromatic phenyl and aliphatic isopropyl) are the crucial factors impacting the diastereoselectivity. Generally, the substrates with bulky R and aromatic phenyl terminal substituent in their allyl groups gave rise to the desired 1,5-dihydro-1,2-azaphosphole 2-oxides **100**, **102**, and **104** in higher diastereoselectivity (Scheme 19) [43]. The RCM reaction is a powerful strategy for the synthesis of *P*-stereogenic 1,5-dihydro-1,2-azaphosphole 2-oxide derivatives, which can be further reduced to 1,2-azaphospholidine 2-oxide derivatives. Thus, the strategy is an efficient method for the synthesis of 1,2-azaphospholidine 2-oxides via the C–C bond formation.

**Scheme 20:**
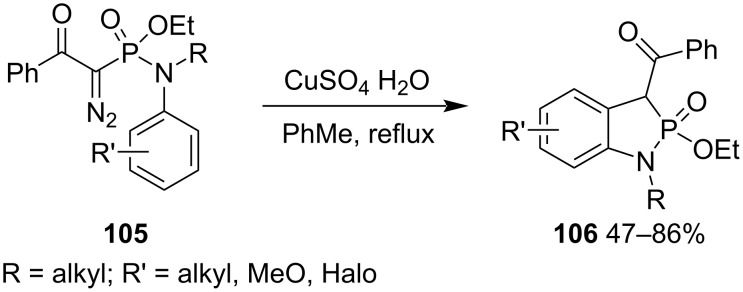
Synthesis of 1-alkyl-3-benzoyl-2-ethoxy-1,3-dihydrobenzo[*d*][1,2]azaphosphole 2-oxides **106** from ethyl *N*-alkyl-*N*-aryl-1-diazo-2-oxo-2-phenylethylphosphonamidates **105**.

Our research group achieved the synthesis of 1-alkyl-3-benzoyl-2-ethoxy-1,3-dihydrobenzo[*d*][1,2]azaphosphole 2-oxide derivatives **106** from ethyl *N*-alkyl-*N*-aryl-1-diazo-2-oxo-2-phenylethylphosphonamidates **105** via the copper-catalyzed intramolecular carbene aromatic C–H bond insertion (Scheme 20) [44]. This is an efficient synthetic strategy for 3-benzoyl-2-ethoxy-1,3-dihydrobenzo[*d*][1,2]azaphosphole 2-oxides **106** through the formation of the C–C bond neighboring at the ring phosphorus atom.

#### Synthesis via formation of the C–C bond neighboring at the ring nitrogen atom

**Scheme 21:**
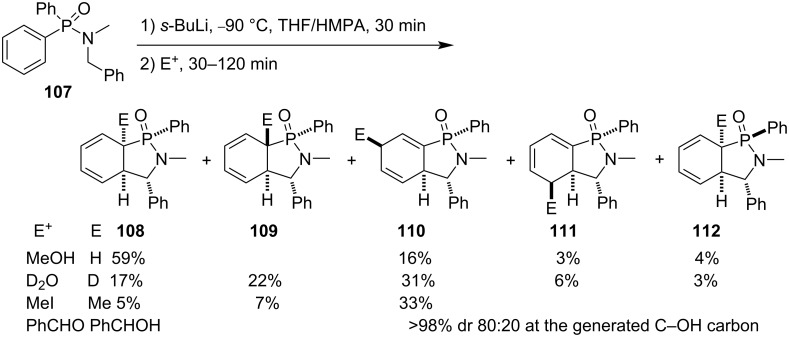
Synthesis of cyclohexadiene-fused γ-phosphinolactams from diphenyl-*N*-benzyl*-N*-methylphosphinamide (**107**).

In 2001, the Ortiz group developed a strategy for the synthesis of γ-phosphinolactam derivatives via formation of the C–C bond neighboring the nitrogen atom. Like the dearomatizing anionic cyclization of *N*-alkyl-*N*-benzylbenzamides [45], the strategy is the dearomatizing anionic cyclization of diaryl-*N*-alkyl-*N*-benzylphosphinamides by treatment with *sec*-butyllithium followed by reactions with different electrophiles, such as water, alcohols, alkyl halides, aldehydes, etc. They first realized the dearomatizing anionic cyclization of diphenyl-*N*-benzyl*-N*-methylphosphinamide (**107**) in the presence of *sec*-butyllithium followed by treatment with methanol, deuterium oxide, methyl iodide, and benzaldehyde, affording a series of cyclohexadiene-fused γ-phosphinolactams **108**–**112** in low regio- and stereoselectivies (Scheme 21) [46].

**Scheme 22:**
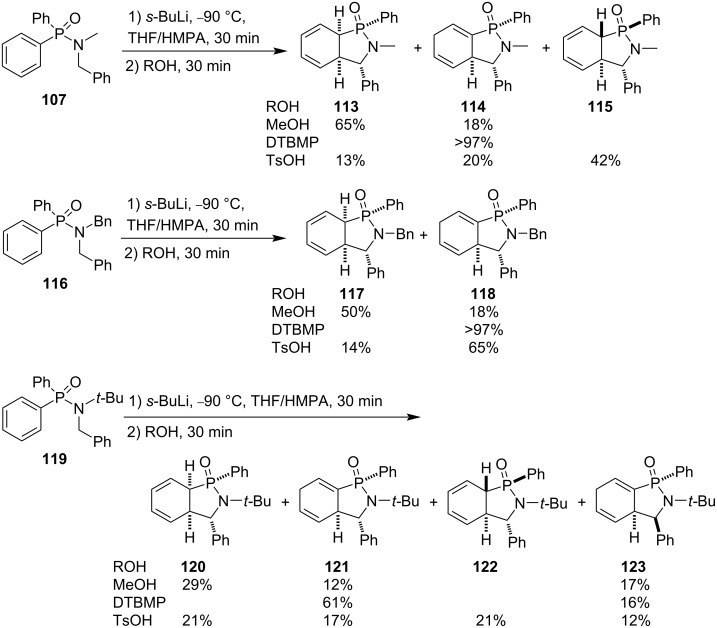
Synthesis of cyclohexadiene-fused γ-phosphinolactams from diphenyl-*N*-alkyl-*N*-benzylphosphinamides.

They further performed a detailed investigation on the dearomatizing anionic cyclization of diphenyl-*N*-alkyl-*N*-benzylphosphinamides **107**, **116**, and **119** by treatment with *sec*-butyllithium and protonation with various alcohols (MeOH, iPrOH, *t*-BuOH), phenols (PhOH, 2-*tert*-butyl-4-methylphenol, and 2,6-di(*tert*-butyl)-4-methylphenol (DTBMP)), trifluoroacetic acid (TFA), and 4-methylbenzenesulfonic acid (TsOH) as the proton donors. The results indicated that the less bulky methanol, more bulky DTBMP, more sterically hindered and acidic TsOH show certain regio- and stereoselectivities, which also depended upon the steric hindrance of the *N*-alkyl group, although several regio- and diastereoisomers were generated in the reactions (Scheme 22) [19].

They further investigated the influences of the time of metalation, the concentration of base, cosolvents (additives), and the time of contact with the electrophiles on the regio- and stereoselectivities in the tandem intramolecular nucleophilic dearomatization of diphenyl-*N*-alkyl-*N*-benzylphosphinamides and reactions with different electrophiles under various reaction conditions [47].

**Scheme 23:**
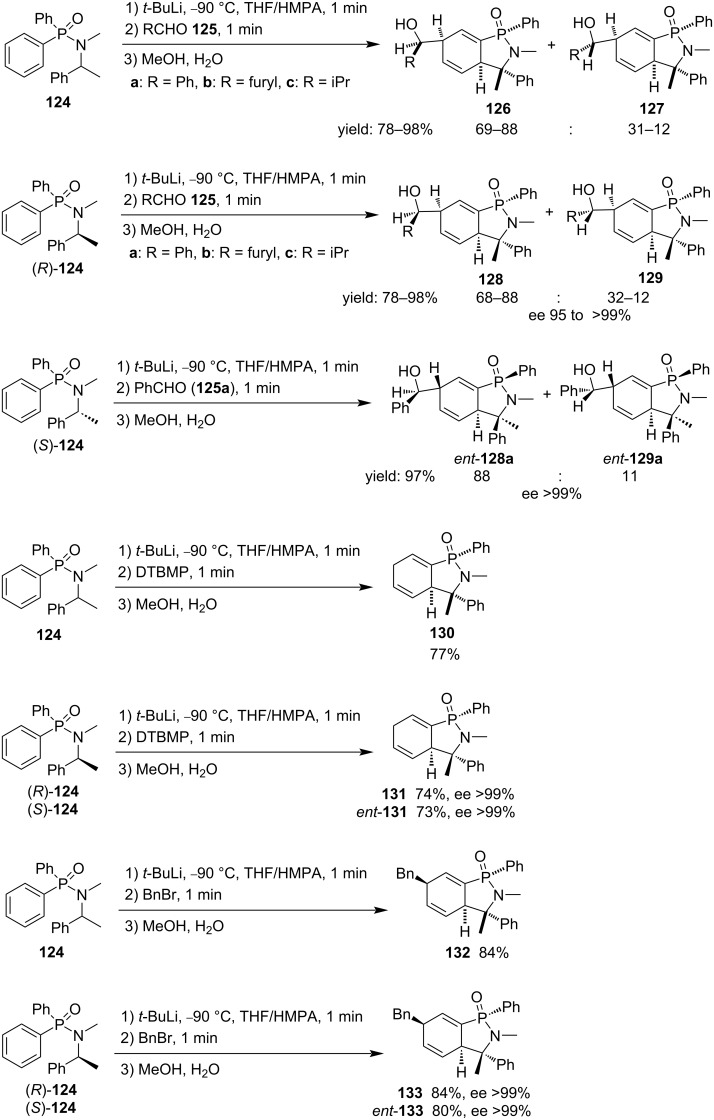
Synthesis of cyclohexadiene-fused γ-phosphinolactams from diphenyl-*N*-methyl-*N*-(1-phenylethyl)phosphinamides **124**.

They used both racemic and enantiopure diphenyl-*N*-methyl-*N*-(1-phenylethyl)phosphinamides **124** as starting materials, realizing an unprecedented asymmetric induction in the synthesis of cyclohexadiene-fused γ-phosphinolactams **126–131**, through the formation of configurationally stable lithium salts. When aldehydes **125** were applied as electrophiles, three new stereocenters were generated in the reaction and the corresponding hydroxymethyl cyclohexadiene-fused γ-phosphinolactams **126–129** were obtained in good to excellent yields with moderate to good diastereoselectivities and excellent enantioselectivities (for both enantiopure starting materials). When DTBMP and benzyl bromide were utilized as electrophiles, only two and one new stereocenter(s) were generated, respectively, and the corresponding protonated and benzylated cyclohexadiene-fused γ-phosphinolactams **130–133** were obtained in good yields, with excellent enantioselectivities for both enantiopure starting materials. In each of the cases, a minor diastereoisomeric epimer in the phosphorus atom was also isolated in less than 8% yield (Scheme 23) [48].

The authors further performed detailed mechanistic investigations experimentally and theoretically [49–50].

**Scheme 24:**
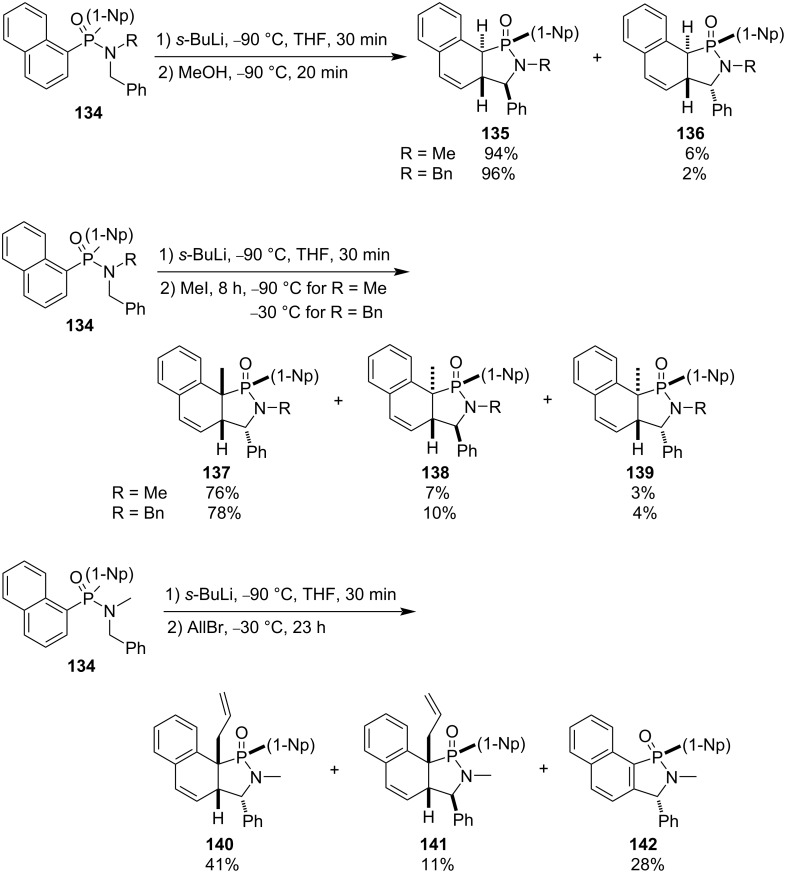
Synthesis of benzocyclohexadiene-fused γ-phosphinolactams from dinaphth-1-yl-*N*-alkyl-*N*-benzylphosphinamides **134**.

Dinaphth-1-yl-*N*-alkyl-*N*-benzylphosphinamides **134** were also applied to the tandem intramolecular nucleophilic dearomatization and protonation or electrophilic alkylation reactions, affording the corresponding dihydronaphthylene-fused γ-phosphinolactams **135–142**. Methanol was used as the electrophile for protonation, while methyl iodide and allyl bromide were used as electrophiles for alkylation. A remarkable difference compared with the diphenylphosphinamides is the fact that the current reactions proceeded with excellent regio- and stereoselectivities and yields in THF without the use of the carcinogenic cosolvent HMPA (Scheme 24) [51].

**Scheme 25:**
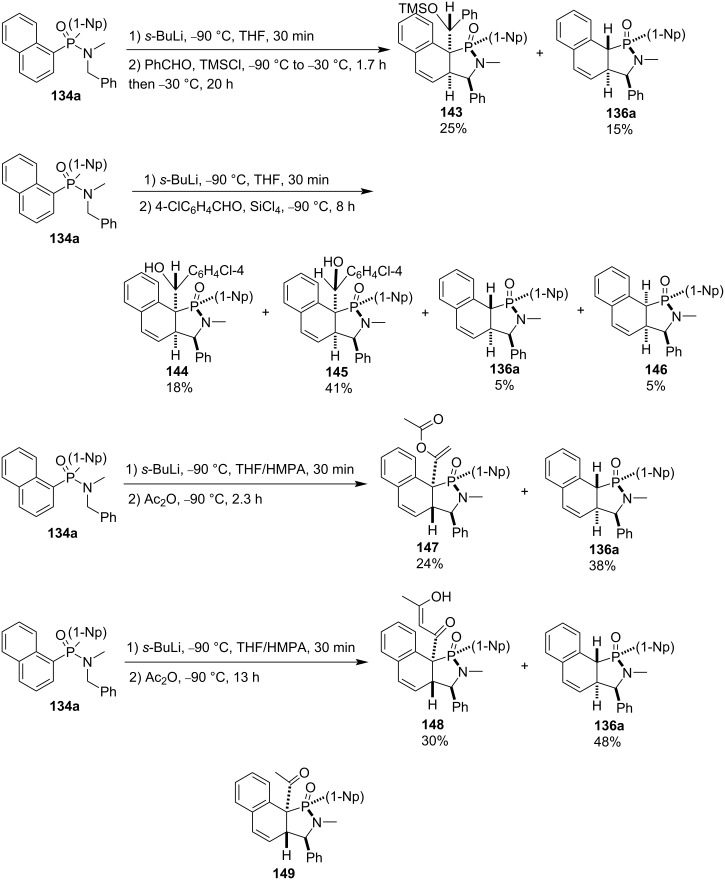
Synthesis of benzocyclohexadiene-fused γ-phosphinolactams from dinaphth-1-yl-*N*-benzyl-*N*-methylphosphinamides.

With benzaldehyde as an electrophile, both alkylated and protonated benzocyclohexadiene-fused γ-phosphinolactams **143** and **136a** were generated in 25% and 15% yield, respectively. However, two pairs of alkylated and protonated benzocyclohexadiene-fused γ-phosphinolactams **144** and **145**, **136a** and **146** were obtained with 4-chlorobenzaldehyde as electrophile. When acetic anhydride was employed as electrophile, besides the protonated product **136a**, two different alkylated products **147** and **148** were formed depending on the reaction time. The 1-acetoxylvinylated and 3-hydroxybut-2-enoylated products **147** and **148** were obtained, respectively, through *O*-acetylation and *C*-acetylation followed by enolization of the first generated acetylated benzocyclohexadiene-fused γ-phosphinolactams **149**. The results indicated that short reaction times favored *O*-acetylation, while long reaction times preferred *C*-acetylation (Scheme 25) [52].

**Scheme 26:**
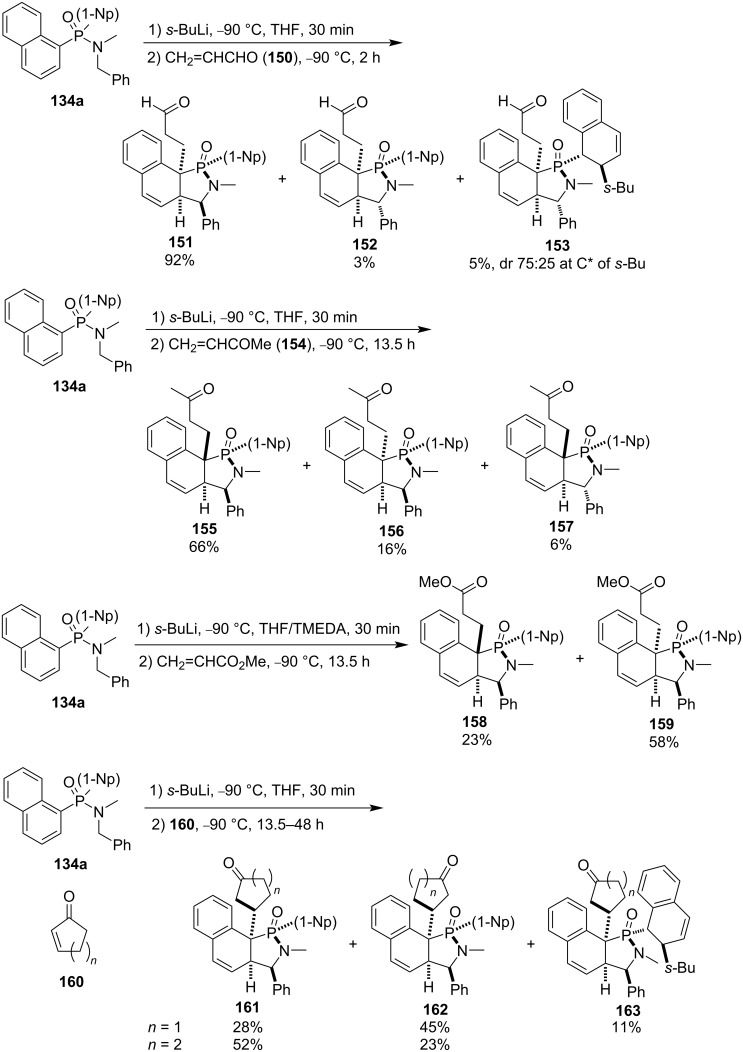
Synthesis of carbonyl-containing benzocyclohexadiene-fused γ-phosphinolactams from dinaphth-1-yl-*N*-benzyl-*N*-methylphosphinamide.

They further investigated various α,β-unsaturated carbonyl compounds as electrophiles. Interestingly, for propenal (**150**), besides two pairs of alkylated products **151** and **152**, the second naphthyl group was alkylated by *sec*-butyllithium, leading to the corresponding product **153** in 5% yield. For but-3-en-2-one (**154**), methyl propenoate, cyclopent-2-enone (**160a**), and cyclohex-2-enone (**160b**), all gave the corresponding diastereomeric alkylated products **155**–**159** and **161**–**163** (Scheme 26) [52].

**Scheme 27:**
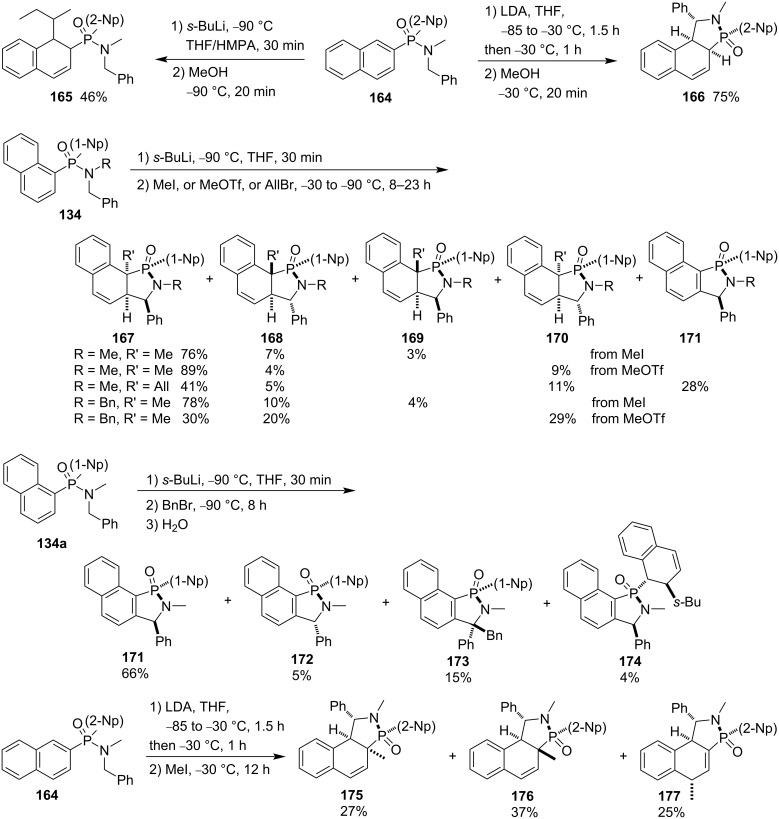
Synthesis of benzocyclohexadiene-fused γ-phosphinolactams from dinaphthyl-*N*-benzyl-*N*-methylphosphinamides.

By contrast, dinaphth-2-yl-*N*-benzyl-*N*-methylphosphinamide (**164**) generated only the *sec*-butylated product **165** by the treatment with *sec*-butyllithium and methanol. However, it gave the corresponding protonated product **166** when LDA was used as base instead of *sec*-butyllithium. Similar as dinaphth-1-yl-*N*-benzyl-*N*-methylphosphinamide (**134a**), the reactions of dinaphth-1-yl-*N*-alkyl-*N*-benzylphosphinamides **134** with but-3-en-2-one generated the corresponding products **167**–**171**. With benzyl bromide as electrophile, dinaphth-1-yl-*N*-benzyl-*N*-methylphosphinamide (**134a**) gave the corresponding diastereomeric products **171**–**173**. The second naphthyl group was alkylated by *sec*-butyllithium, affording product **174** in 4% yield as well. In the presence of LDA as base instead of *sec*-butyllithium and with MeI as electrophile, dinaphth-2-yl-*N*-benzyl-*N*-methylphosphinamide (**164**) gave three pairs of methylated diastereomeric products **175**–**177** (Scheme 27) [53].

**Scheme 28:**
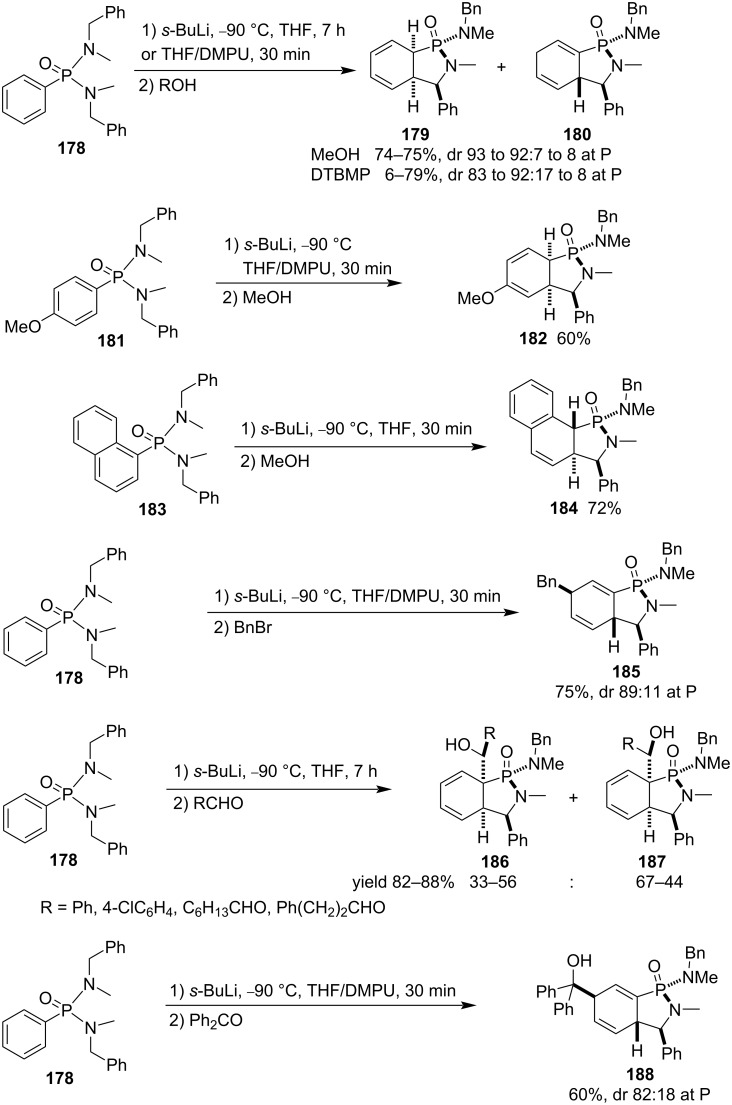
Synthesis of cyclohexadiene-fused 1-(*N*-benzyl-*N*-methyl)amino-γ-phosphinolactams from aryl-*N,N’*-dibenzyl-*N,N*’-dimethylphosphonodiamides.

They also explored the application of their strategy in the dearomatizing cyclization of aryl-*N,N’*-dibenzyl-*N,N*’-dimethylphosphonodiamides **178**, **181**, and **183**. The corresponding cyclohexadiene-fused γ-phosphinolactam derivatives **179**, **180**, **182**, and **184**–**188** were obtained after treatment with *sec*-butyllithium in a mixture of THF and DMPU (1,3-dimethyl-3,4,5,6-tetrahydro-2-pyrimidinone) followed by addition of various electrophiles, such as methanol, phenolic DTBMP, benzyl bromide, aldehydes, and benzophenone. For both benzyl bromide and benzophenone, their alkylations occurred only at the γ-position of the phenyl group in phenylphosphonodiamides due to less bulkyness (Scheme 28) [54].

**Scheme 29:**
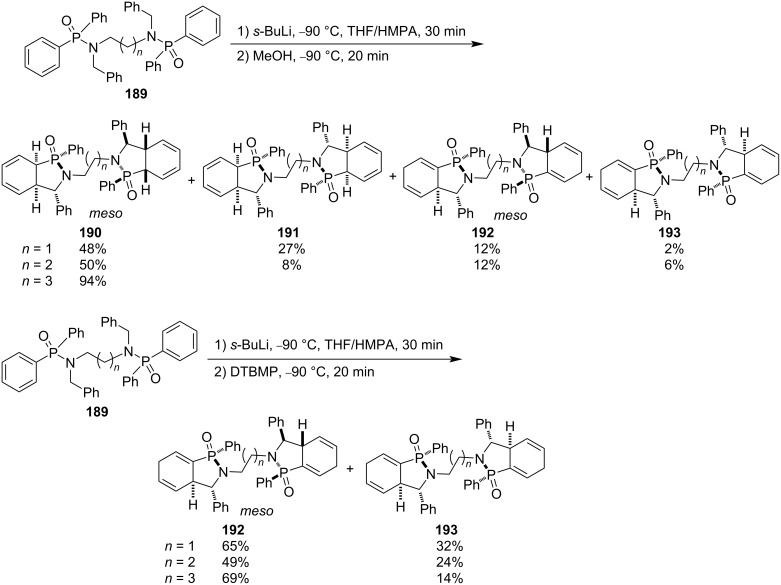
Synthesis of bis(cyclohexadiene-fused γ-phosphinolactam)s from bis(diphenyl-*N*-benzylphosphinamide)s.

They finally extended their strategy to alkyl-linked bis(diphenyl-*N*-benzylphosphinamide)s **189**. Both pairs of *meso*-bis(cyclohexadiene-fused γ-phosphinolactam)s **190** and **192**, **190** as major products, and two pairs of racemic diastereomeric bis(cyclohexadiene-fused γ-phosphinolactam)s **191** and **193** were obtained when the reactions were quenched with less steric methanol as the electrophile because the protonation occurred at both the α- and γ-positions of the phenyl group in their diphenylphosphinamide moieties. However, only one pair of *meso*-bis(cyclohexadiene-fused γ-phosphinolactam)s **192** as major products and one pair of racemic diastereomeric bis(cyclohexadiene-fused γ-phosphinolactam)s **193** were obtained when the reactions were quenched with more bulky DTBMP as the electrophile due to protonation at only the less bulky γ-position of the phenyl group in their diphenylphosphinamide moieties (Scheme 29) [55].

**Scheme 30:**
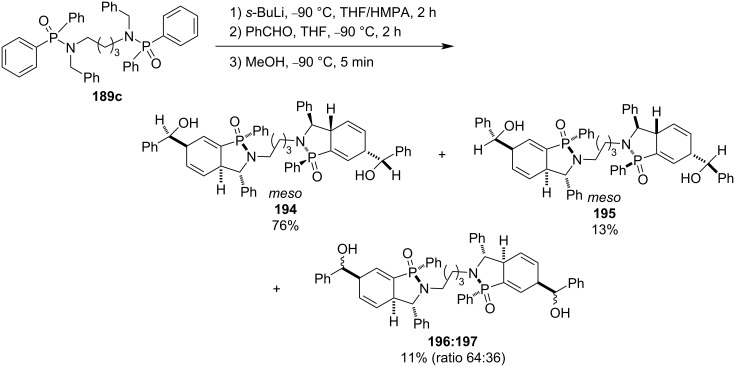
Synthesis of bis(hydroxymethyl-derived cyclohexadiene-fused γ-phosphinolactam)s from tetramethylene-linked bis(diphenyl-*N*-benzylphosphinamide).

When tetramethylene-linked bis(diphenyl-*N*-benzylphosphinamide) (**189c**, *n* = 3) was treated with *sec*-butyllithium followed by addition of benzaldehyde and methanol sequentially, the reaction afforded four diastereomeric tetramethylene-linked bis(hydroxymethyl-derived cyclohexadiene-fused γ-phosphinolactam)s **194**–**197** generated by alkylation at the γ-position of the phenyl group in their diphenylphosphinamide moieties due to favorable steric hindrance. One pair (**194**) of *meso*-diastereomers is the major product (Scheme 30) [55].

### Synthesis of 1,2-azaphospholidine 2-oxide derivatives via annulations

Annulations are alternative strategies for the construction of 1,2-azaphospholidine 2-oxides and their fused derivative, [4 + 1] annulations and more occasionally a [3 + 2] annulation.

#### [4 + 1] Annulation via formations of both C–N and N–P bonds

**Scheme 31:**
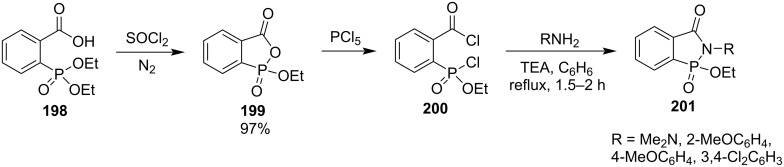
Synthesis of 2-aryl/dimethylamino-1-ethoxy-2-hydrobenzo[*c*][1,2]azaphosphol-3-one 1-oxides from ethyl (2-(chlorocarbonyl)phenyl)phosphonochloridate **200** with amines and *N,N*-dimethylhydrazine.

Miles and Street prepared 2-aryl/dimethylamino-1-ethoxy-2-hydrobenzo[*c*][1,2]azaphosphol-3-one 1-oxides **201** from 2-(diethoxyphosphoryl)benzoic acid (**198**) and amine derivatives. They first converted 2-(diethoxyphosphoryl)benzoic acid (**198**) into its anhydride 1-ethoxy-3*H*-benzo[*c*][1,2]oxaphosphol-3-one 1-oxide (**199**) by treatment with thionyl chloride under nitrogen. The mixed anhydride **199** was further treated with phosphorus pentachloride to generate ethyl (2-(chlorocarbonyl)phenyl)phosphonochloridate (**200**), which reacted with amine and hydrazine derivatives in the presence of triethylamine, affording the desired 1-ethoxy-2-hydrobenzo[*c*][1,2]azaphosphol-3-one 1-oxides **201** but the yields were not reported (Scheme 31) [56].

**Scheme 32:**
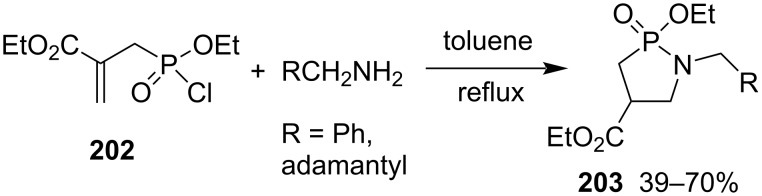
Synthesis of ethyl 2-ethoxy-1,2-azaphospholidine-4-carboxylate 2-oxides from ethyl 2-((chloro(ethoxy)phosphoryl)methyl)acrylate (**202**) and primary amines.

Two ethyl 1-substituted 2-ethoxy-1,2-azaphospholidine-4-carboxylate 2-oxides **203** were synthesized in 70% and 39% yields, respectively, from ethyl 2-((chloro(ethoxy)phosphoryl)methyl)acrylate (**202**) and benzyl- and adamantylmethylamines via aza-Michael addition and intramolecular nucleophilic substitution. The synthetic method showed very limited substrate scope. Only less bulky primary amines underwent the first aza-Michael addition and then intramolecular nucleophilic substitution. However, aromatic amines, aniline, 2,3-dihydro-1*H*-inden-4-amine, and the bulky aliphatic primary amine adamantylamine did not proceed due to their reduced nucleophilicities, generating the corresponding phosphonamidates only through the reaction with the more electrophilic phosphonochloridate (Scheme 32) [57].

#### [4 + 1] Annulation via formations of both C–P and P–N bonds

**Scheme 33:**
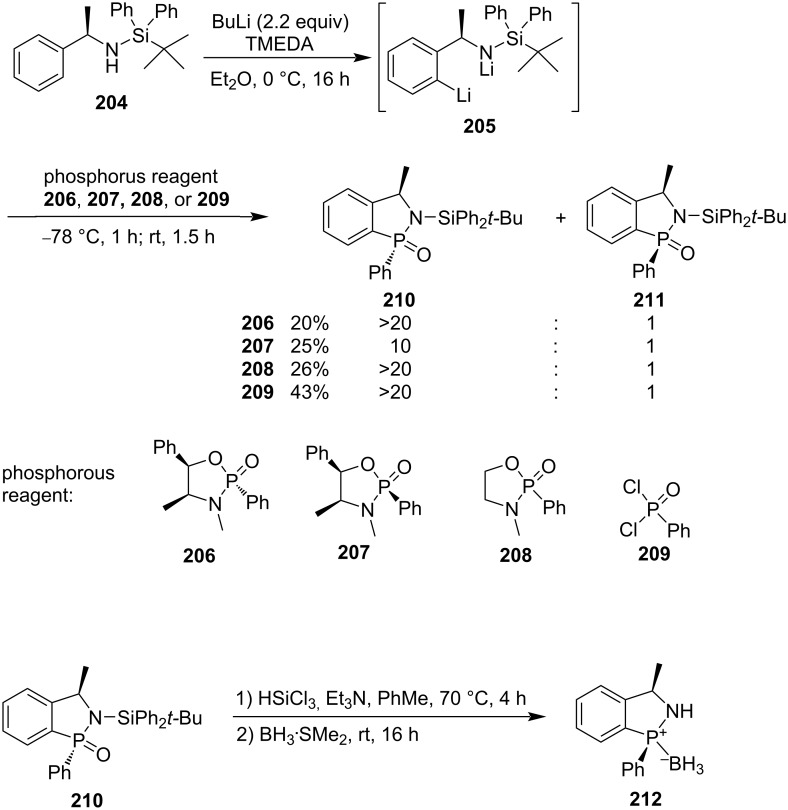
Synthesis of (1*S*,3*R*)-2-(*tert*-butyldiphenylsilyl)-3-methyl-1-phenyl-2,3-dihydrobenzo[*c*][1,2]azaphosphole 1-oxide via double displacement of phosphorus electrophiles with (*R*)-1-*tert*-butyl-1,1-diphenyl-*N*-(1-phenylethyl)silanamine (**204**).

(1*S*,3*R*)-2-(*tert*-Butyldiphenylsilyl)-3-methyl-1-phenyl-2,3-dihydrobenzo[*c*][1,2]azaphosphole 1-oxide (**210**) was synthesized in low yields of 20–43% with diastereomeric ratios of 10:1 to >20:1 from (*R*)-1-*tert*-butyl-1,1-diphenyl-*N*-(1-phenylethyl)silanamine (**204**) via the treatment with butyllithium followed by double displacement with four different phosphorus electrophiles **206**–**209**, which include three 1,3,2-oxazaphospholidine 2-oxide derivatives **206**–**208** and phenylphosphonic dichloride (**209**). The product **210** was further transformed into (1*S*,3*R*)-3-methyl-1-phenyl-2,3-dihydro-1*H*-benzo[*c*][1,2]azaphosphole **212**, which can be applied as a precatalyst. However, its borane complex showed 23% enantiomeric excess in the asymmetric borane reduction of acetophenone in THF at room temperature (Scheme 33) [58].

**Scheme 34:**
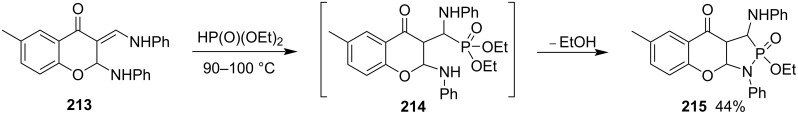
Synthesis of 2,3,3a,9a-tetrahydro-4*H*-1,2-azaphospholo[5,4-*b*]chromen-4-one (**215**) from 3-(phenylaminomethylene)-2-phenylamino-6-methyl-2,3-dihydro-4*H*-chromen-4-one (**213**) and diethyl phosphite.

The reaction of 3-(phenylaminomethylene)-2-phenylamino-6-methyl-2,3-dihydro-4*H*-chromen-4-one (**213**) and diethyl phosphite at 90–100 °C generated 2-ethoxy-6-methyl-2-oxo-1-phenyl-3-phenylamino-2,3,3a,9a-tetrahydro-4*H*-1,2-azaphospholo[5,4-*b*]chromen-4-one (**215**) in 44% yield through the Michael addition and subsequent intramolecular aminolysis (Scheme 34) [15]. The products are potential anti-inflammatory agents.

**Scheme 35:**
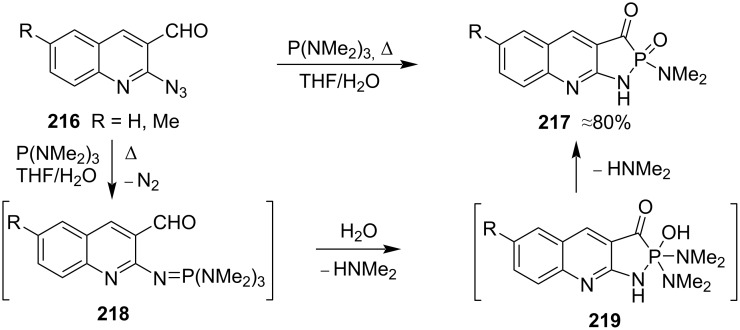
Synthesis of quinoline-fused 1,2-azaphospholine 2-oxides from 2-azidoquinoline-3-carbaldehydes and tris(dimethylamino)phosphine.

In 2013, to develop potent anti-inflammatory agents, Abdou and co-workers prepared quinoline-fused 1,2-azaphospholine 2-oxides **217** in approximate 80% yield from 2-azidoquinoline-3-carbaldehydes **216** and tris(dimethylamino)phosphine in THF with water as solvent. It was mentioned that 2-azidoquinoline-3-carbaldehyde **216** and tris(dimethylamino)phosphine first generated phosphinimines **218** followed by quenching with water and sequential loss of two molecules of dimethylamine. However, the mechanism of this transformation was not clearly investigated (Scheme 35) [16].

**Scheme 36:**
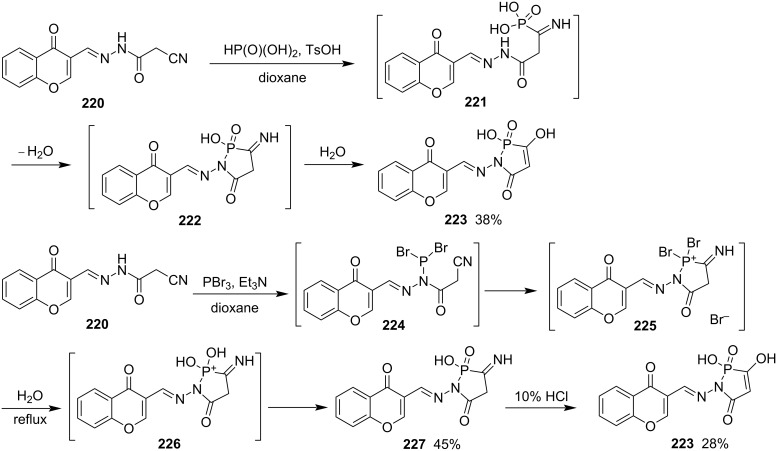
Synthesis of 1-hydro-1,2-azaphosphol-5-one 2-oxide from cyanoacetohydrazide with phosphonic acid and phosphorus tribromide.

To develop new antitumor agents composed of chromene and 5-oxo-1,2-azaphospolidine 2-oxide motifs, Ali’s group performed the reaction of (*E*)-2-cyano-*N*'-((4-oxo-4*H*-chromen-3-yl)methylene)acetohydrazide (**220**) and phosphonic acid in the presence of 4-toluenesulfonic acid in dioxane, affording (*E*)-2,3-dihydroxy-1-(((4-oxo-4*H*-chromen-3-yl)methylene)amino)-1-hydro-1,2-azaphosphol-5-one 2-oxide (**223**) in 38% yield via the acid-catalyzed addition to the cyano group and subsequent cyclocondensation, hydrolysis, and tautomerization. Alternatively, the reaction of (*E*)-2-cyano-*N*'-((4-oxo-4*H*-chromen-3-yl)methylene)acetohydrazide (**220**) and phosphorus tribromide in the presence of triethylamine in dioxane followed by two step hydrolysis gave the same product **223** in 28% yield. Phosphorus tribromide first reacted with more nucleophilic amide nitrogen atom to form dibromophosphanamine derivative **224** by loss of HBr. The more nucleophilic dibromophosphanamine derivative **224** further underwent a nucleophilic addition to the cyano group followed by hydrolysis to give the final product **223** (Scheme 36). The product **223** showed antitumor activity in a biological assay [20].

**Scheme 37:**
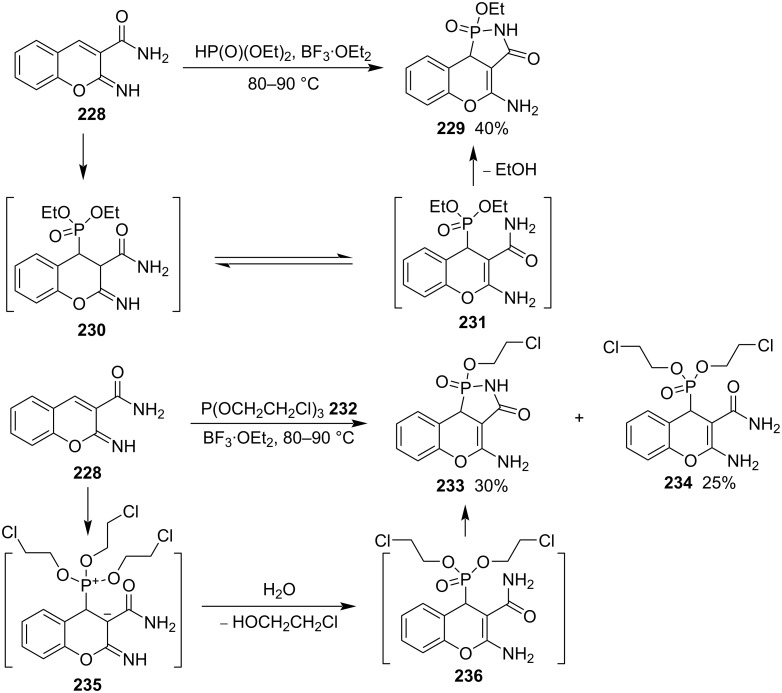
Synthesis of chromene-fused 5-oxo-1,2-azaphospolidine 2-oxides.

To develop antioxidants and antitumor agents, the same group synthesized chromene-fused 5-oxo-1,2-azaphospholidine 2-oxide derivatives. The reaction of 2-imino-2*H*-chromene-3-carboxamide (**228**) and diethyl phosphite at 80–90 °C under the catalysis of boron trifluoride, afforded 4-amino-1-ethoxy-9b-hydrochromeno[4,3-*c*][1,2]azaphosphol-3(2*H*)-one 1-oxide (**229**) in 40% yield through the Michael addition and subsequent tautomerization and intramolecular aminolysis. Under similar conditions, the reaction of 2-imino-2*H*-chromene-3-carboxamide (**228**) and tris(2-chloroethyl) phosphite (**232**) generated 4-amino-1-(2-chloroethoxy)-9b-hydrochromeno[4,3-*c*][1,2]azaphosphol-3(2*H*)-one 1-oxide (**233**) in 30% yield through the Michael addition and subsequent tautomerization and intramolecular aminolysis. Non-aminolysis byproduct **234** was obtained in 25% (Scheme 37). Both of the chromene-fused 5-oxo-1,2-azaphospholidine 2-oxide derivatives **233** and **234** showed good antioxidant and antitumor activities [17].

#### [4 + 1] Annulation via formations of both C–C and C–N bonds

**Scheme 38:**
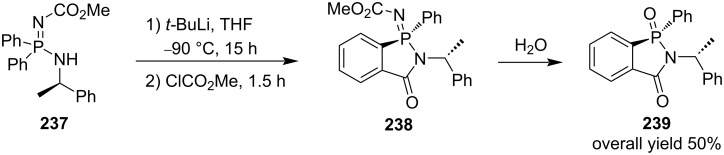
Synthesis of (*R*)-1-phenyl-2-((*R*)-1-phenylethyl)-2-hydrobenzo[*c*][1,2]azaphosphol-3-one 1-oxide (**239**) from methyl (*R*)-(diphenyl((1-phenylethyl)amino)-λ^5^-phosphanylidene)carbamate (**237**) via *ortho*-directed lithiation followed by electrophilic quench and intramolecular aminolysis.

When the Ortiz group investigated the directed *ortho*-lithiation of aminophosphazenes, they realized one example synthesis of (*R*)-1-phenyl-2-((*R*)-1-phenylethyl)-2-hydrobenzo[*c*][1,2]azaphosphol-3-one 1-oxide (**239**) from methyl (*R*)-(diphenyl((1-phenylethyl)amino)-λ^5^-phosphanylidene)carbamate (**237**) via the *ortho*-directed lithiation with *tert*-butyllithium with carbamate as the directing group followed by electrophilic quench with methyl chloroformate and intramolecular aminolysis. Finally, hydrolysis removed the directing group, affording the final product **239** in 50% yield (Scheme 38) [59].

**Scheme 39:**
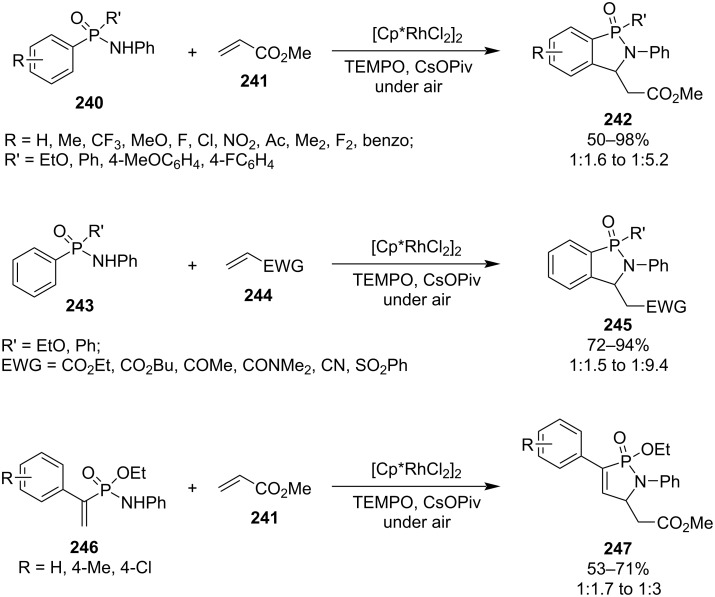
Synthesis of dihydro[1,2]azaphosphole 1-oxides from aryl/vinyl-*N*-phenylphosphonamidates and aryl-*N*-phenylphosphinamides with electron-withdrawing ethenes via the rhodium-catalyzed oxidative coupling and subsequent intramolecular aza-Michael addition.

Structurally diverse methyl 2-(1-ethoxy-1-oxido-2-phenyl-2,3-dihydrobenzo[*c*][1,2]azaphosphol-3-yl)acetates **242a** (R’ = OEt) and methyl 2-(1-aryl-1-oxido-2-phenyl-2,3-dihydrobenzo[*c*][1,2]azaphosphol-3-yl)acetates **242b**–**d** (R’ = Ar) were prepared in 50–98% yield with diastereomeric ratio of 1:1.6–1:5.2 from ethyl aryl-*N*-phenylphosphonamidates **240a** (R’ = OEt) and diaryl-*N*-phenylphosphinamides **240b–d** (R’ = Ar), respectively, with methyl acrylate (**241**) via the rhodium-catalyzed oxidative coupling and subsequent intramolecular aza-Michael addition. Methyl acrylate (**241**) could be replaced by various electron-deficient olefins **244**, including ethyl and butyl acrylates, but-3-en-2-one, *N*,*N*-dimethylacrylamide, acrylonitrile, and phenyl vinyl sulfone. In addition, ethyl 1-arylvinyl-*N*-phenylphosphonamidates **246** were able to react with methyl acrylate (**241**), affording methyl 2-(3-aryl-2-ethoxy-2-oxo-1-phenyl-1,5-dihydro-1,2-azaphosphol-5-yl)acetates **247** in moderate yields of 53–71% with diastereomeric ratios of 1:1.7 to 1:3. The synthetic strategy is more versatile (Scheme 39) [60].

#### [3 + 2] Annulation via formations of both C–C and P–N bonds

**Scheme 40:**
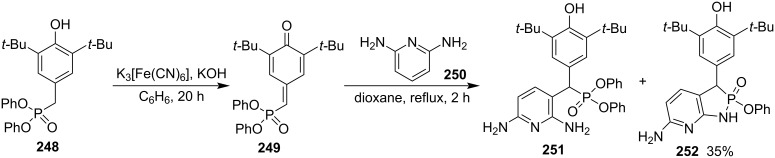
Synthesis of 1,3-dihydro-[1,2]azaphospholo[5,4-*b*]pyridine 2-oxides.

After oxidation with K_3_Fe(CN)_6_, diphenyl 3,5-di(*tert*-butyl)-4-hydroxybenzylphosphonate (**248**) was oxidized into diphenyl (3,5-di-*tert*-butyl-4-oxocyclohexa-2,5-dienylidene)methylphosphonate (**249**), which was reacted with 2,6-diaminopyridine (**250**) in acetonitrile at room temperature to give diphenyl 3,5-di(*tert*-butyl)-4-hydroxyphenyl-(2,6-diaminopyridin-3-yl)methylphosphonate (**251**) with 6-amino-3-(3,5-di-*tert*-butyl-4-hydroxyphenyl)-2-phenoxy-1,3-dihydro-[1,2]azaphospholo[5,4-*b*]pyridine 2-oxide (**252**) as byproduct. The yield of 6-amino-3-(3,5-di-*tert*-butyl-4-hydroxyphenyl)-2-phenoxy-1,3-dihydro-[1,2]azaphospholo[5,4-*b*]pyridine 2-oxide (**252**) was improved to 35% when the reaction was conducted in refluxing dioxane for 2 h. It was generated from the intramolecular aminolysis of diphenyl 3,5-di(*tert*-butyl)-4-hydroxyphenyl-(2,6-diaminopyridin-3-yl)methylphosphonate (**251**) and showed excellent antioxidant activity (Scheme 40) [18].

## Conclusion

1,2-Azaphospholidine 2-oxides and 1,2-azaphospholine 2-oxides are important five-membered azaphosphaheterocycles, They are known as γ-phostams, including γ-phosphonolactams and γ-phosphinolactams. Benzo[1,2]azaphospholine 2-oxides are phosphorus analogues of indolinone derivatives and show important biological activities. Both γ-phosphonolactams and γ-phosphinolactams and their fused derivatives have been synthesized through various synthetic strategies. The synthetic strategies can be categorized into cyclization and annulation strategies. The cyclizations have been widely applied for the formation of any C–N, C–P, P–N, and C–C bonds in the 1,2-azaphospholidine ring. [4 + 1] Annulations have been mainly utilized in the construction of the 1,2-azaphospholidine ring, while the [3 + 2] annulation has been seldomly used in the synthesis of pyridine-fused 1,2-azaphospholidine 2-oxide only. Few asymmetric synthetic methods have been developed to date. Thus, highly stereoselective asymmetric synthetic methods to access 1,2-azaphospholidine 2-oxides and 1,2-azaphospholine 2-oxides, and their fused and spiro derivatives are in high demand and should be developed in the near future.
